# Increased detection of enterovirus A71 infections, Germany, 2019

**DOI:** 10.2807/1560-7917.ES.2019.24.39.1900556

**Published:** 2019-09-26

**Authors:** Sindy Böttcher, Sabine Diedrich, Kathrin Keeren, the Laboratory Network for Enterovirus Diagnostic (LaNED)

**Affiliations:** 1 National Reference Centre for Poliomyelitis and Enteroviruses, Robert Koch Institute, Berlin, Germany; 2 Secretary of the National Commission for Polio Eradication in Germany, Robert Koch Institute, Berlin, Germany; 3 The members of the network are listed at the end of the article

**Keywords:** Enterovirus A71, enterovirus, surveillance, viral meningitis, Germany

## Abstract

We report on the increased circulation of enterovirus A71 in Germany in 2019. Strains were mainly identified in hospitalised patients with suspected aseptic meningitis/encephalitis. Molecular analysis showed co-circulation of EV-A71 sub-genogroups C1 and C4, a signal for physicians and public health authorities to include/intensify EV diagnostic in patients showing signs of aseptic meningitis, encephalitis or acute flaccid paralysis/myelitis.

In Europe, enterovirus A71 (EV-A71) has mainly been detected sporadically in patients with neurological disorders. C2 has been the predominant sub-genogroup during the last decade [1]. A multi-recombinant C1 lineage (C1-like) emerged in 2015 in Germany [2-4]. Since then, it was detected in sporadic cases with severe neurological disease in several European countries [4-6], as well as in an outbreak in Catalonia, Spain in 2016 [7,8]. In south-eastern Asia, EV-A71, sub-genogroup C4 strains have been mainly associated with severe and even fatal courses of hand, foot and mouth disease (HFMD) in children [1].

Here we report on an increase of EV-A71 C1-like strains in Germany in 2019 and first detections of EV-A71 C4 strains in patients with neurological disease and HFMD.

## Enterovirus surveillance in Germany

In Germany, enterovirus surveillance (EVSurv) is conducted within the framework of the Global Polio Eradication Initiative and provides continuous data on circulating enteroviruses since 2006. Stool or cerebrospinal fluid samples of hospitalised patients with signs of aseptic meningitis/encephalitis and/or acute flaccid paralysis are analysed within a quality-controlled laboratory network (LaNED). Laboratory results and pseudonymised patient data are reported to the national public health institute (Robert Koch Institute; RKI). Data are analysed and the results are publically available at evsurv.rki.de. Previous data on EV-A71 circulation suggested that EV-A71 strains circulate in upsurges every 3 years, with 2010 and 2013 being epidemic years in Germany [2].

Typing of enterovirus (EV) strains is performed according to the individual network laboratory algorithm, by sequencing the complete or partial VP1 or VP4/VP2 region and/or virus isolation and typing by neutralisation assay. Assignment of the EV-A71 strains to one of the seven known sub-genogroups was performed using the Enterovirus Genotyping Tool [9] and/or neighbour-joining algorithm-based phylogenetic tree analysis. All sequences were submitted to GenBank under accession numbers (MN397830-MN397906).

Since 2006, ca 2,500 samples have been tested annually within the EVSurv for a total of ca 34,000 analysed since that time. Of these, 25–30% were EV-positive. Overall, echovirus 30 was the most prevalent EV type (n = 2,194), followed by echovirus 6 (n = 775) and EV-A71 (n = 496). A total of 32 EV-A71 positive samples were documented in 2017 and 40 in 2018.

## Enterovirus A71 detections through enterovirus surveillance

From January 2019 to the end July 2019, 38 EV-A71 positive patients were registered, including one fatal case. EV-A71-positive patients were observed in March (n = 2), April (n = 3), May (n = 6), June (n = 14) and July (n = 13). Besides EV-A71, CV-B5 was the second most common EV type (n = 24) within the EVSurv this year. Typing results are still pending for 35% (73/209) of EV-positive samples.

Of the 38 EV-A71 strains detected, 26 were analysed further at the National Reference Centre for Poliomyelitis and Enteroviruses (NRZ PE). Sequencing of the VP1 region was done as described previously [2]. Twenty-one strains were assigned to C1-like, four to sub-genogroup C4 and one to sub-genogroup C2 (Table 1).

**Table 1:** Number of enterovirus surveillance samples analysed and enterovirus A71 subtyping results, Germany, 2016–July 2019

EVSurv samples tested	2016 (n)	2017 (n)	2018 (n)	Jan–Jul 2019 (n)
Total	2,444	2,188	1,959	1,160
EV-positive	527	448	416	209
EV-typed	445	380	349	136
EV-A71-positive	77	32	40	38
**EV-A71 sub-genogroups**				
Lineage C1-like	57	18	23	21
C2	10	6	0	1
C4	0	0	12	4
ND	10	8	5	12

EV: enterovirus; EVSurv: enterovirus surveillance; Jan: January; Jul: July; ND: not determined.

Number of EV-A71 strains and sub-genogroup assignment based on the VP1 region is shown.

### Epidemiological data

EV-A71-positive samples were reported from 11 of 16 federal states in Germany, with patients hospitalised in 20 different hospitals. All patients were between 0 and 10 years old, with 30 of 38 between 0 and 5 years old. Of these, 8 were younger than 1 year old. Twenty-one were male and 17 were female. All EV-A71 strains were detected in stool samples.

### Phylogenetic relation

**Figure 1:**
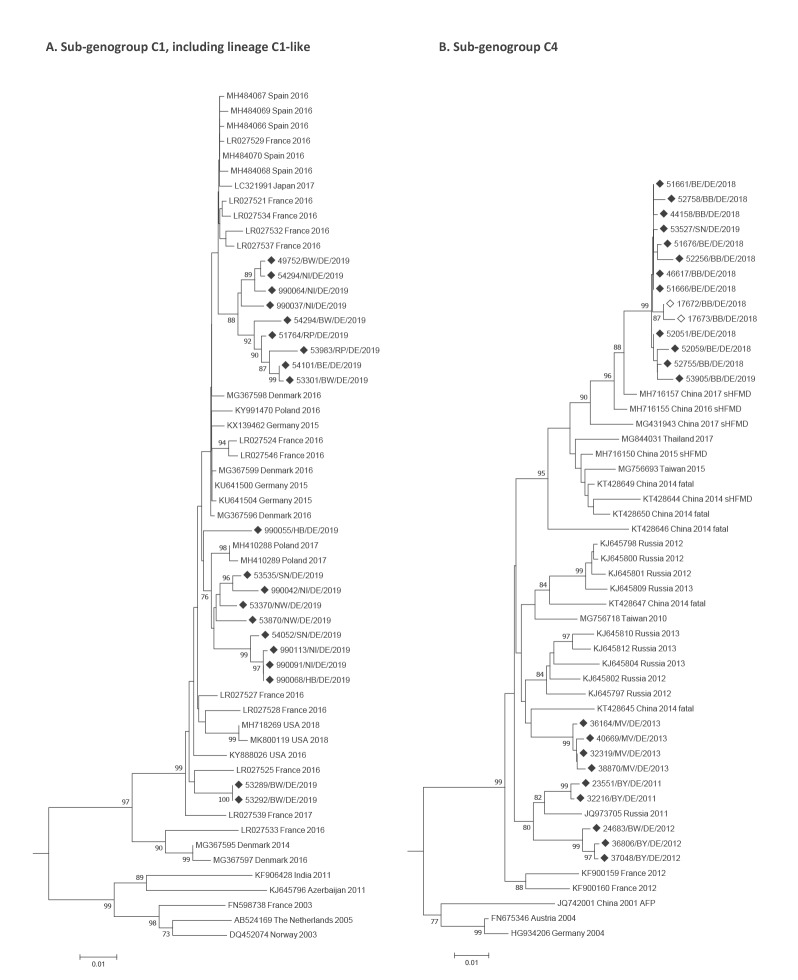
Phylogenetic trees of (A) sub-genogroup C1, including lineage C1-like and (B) sub-genogroup C4, 2019

Sequence analysis of C1-like strains identified in 2019 showed high nucleotide (nt) identities with recently detected strains, indicating a rapid widespread circulation of this new recombinant variant [4]. Phylogenetic tree calculation showed several lineages either grouping separately or clustering to recent strains detected in France, Poland, Spain, Denmark, the United States (US) and Japan (Figure 1A).

The phylogenetic tree is based on complete viral protein 1 (VP1) nucleotide (nt) sequences (891 b corresponding to nt position 2439–3329 in the prototype BrCr ETU22521). Strains identified within the German enterovirus surveillance (EVSurv) and a representative set of enterovirus A71 strains retrievable from GenBank were used. Only sequences covering the complete VP1 region were included in the analysis. Subtrees including only EV-A71 sub-genogroup C1, including lineage C1-like (A) and EV-A71 sub-genogroup C4 (B) are shown. Strains identified within the EVSurv are marked with filled diamonds and strains identified in hand, foot and mouth disease (HFMD) patients with open diamonds. The tree was calculated using the neighbour-joining algorithm with 1,000 replicates in MEGA software version 7.0.26. Only bootstrap values > 70 are shown. Scale bars indicate nt substitution per site.

Sequence analysis of sub-genogroup C4 showed separate clustering of the German EV-A71 C4 strains detected in 2011–2013 and 2018–July 2019. Currently-circulating strains grouped together with EV-A71 C4 strains detected in south-eastern Asia (Figure 1B) with highest nt identities to MH716155, MH716157 and MG431943 detected in patients with severe HFMD. This cluster also includes strains isolated from patients with severe HFMD and even fatal courses (KT428649, KT428650).

## Enterovirus A71 detections in routine samples

The increased circulation of EV-A71 in 2019 has also been indicated by samples routinely sent to the NRZ PE, mostly from other laboratories sending in EV-positive samples for typing, but also from clinicians/hospitals not yet participating in EVSurv or those submitting samples from patients with diseases other than suspected aseptic meningitis/encephalitis or acute flaccid paralysis/myelitis. Of 126 EV strains typed from January through July 2019, 23 were assigned to EV-A71. Of these, 19 were assigned to EV-A71 lineage C1-like, three to EV-A71 sub-genogroup C4 and one to EV-A71 sub-genogroup C2 (Table 2). Notably, the number of EV-A71 detections in the first 7 months of 2019 (n = 23) has already exceeded the total of such detections for 2017 and 2018, as well as the totals from the epidemic years of 2010 (6/61), 2013 (12/206) and 2016 (17/144).

**Table 2:** Number of samples sent to the National Reference Centre for Poliomyelitis and Enteroviruses for enterovirus detection and typing, Germany, 2010–July 2019

NRZ PE samples tested	2010 (n)	2011 (n)	2012 (n)	2013 (n)	2014 (n)	2015 (n)	2016 (n)	2017 (n)	2018 (n)	Jan–Jul 2019 (n)
Total	128	78	137	279	166	153	252	279	278	213
EV-positive	61	58	59	206	93	81	144	147	181	126
EV-A71 positive	6	1	7	12	2	12	17	6	7	23
**EV-A71 sub-genogroup**										
C1	0	0	1	3	1	0	0	0	0	0
Lineage C1-like	0	0	0	0	0	7	9	2	7	19
C2	6	1	1	9	0	4	8	4	0	1
C4	0	0	5	0	1	1	0	0	0	3

EV: enterovirus; Jan: January; Jul: July; NRZ-PE: National Reference Centre for Poliomyelitis and Enteroviruses.

Number of EV-A71 strains and sub-genogroup assignment based on the VP1 region is shown.

Germany does not conduct standardised HFMD surveillance, but occasionally, samples collected within nursery school outbreaks of HFMD are submitted to the NRZ PE by the local health authorities. From 2010 until 2018, between 15 and 82 samples from HFMD patients were submitted per year, with 6 to 57 patients being positive for EV and 2 to 8 patients being positive for EV-A71 annually. In 2018, there were 24 HFMD patients positive for EV, eight of whom had EV-A71. Of those eight, EV-A71 C4 was detected for the first time in four children with HFMD. For two strains (17672, 17673), the VP1 region was sequenced and compared with EV-A71 C4 strains identified in samples analysed within the EVSurv in 2018 and 2019. Close clustering indicates that there is no relationship between the VP1 region sequence and the presence of HFMD or symptoms related to aseptic meningitis/encephalitis (Figure 1B).

## Discussion and conclusion

Our findings suggest an increased circulation of EV-A71 strains in Germany already in the first 7 months of the year. Considering the fact that the EV season is still ongoing, the data available suggest that 2019 will become an epidemic year similar to 2016, 2013 and 2010.

The emergence of new EV variants exhibiting altered antigenic sites might result in increased circulation and/or changed clinical manifestation. In Europe, EV-A71 was only sporadically associated with neurological disease during the past 30 years. In Germany, the predominant EV-A71 sub-genogroup C2 was replaced by C1-like strains in 2016. Emergence of EV-A71 sub-genogroup C4 strains in Germany was detected between 2011 and 2013 [2], with France [10], Russia [11] and Denmark [12] also reporting such detection around the same time.

One limitation of the EVSurv is that no detailed clinical information is available since the EVSurv request form deliberately only asks for basic cardinal symptoms: fever, nuchal rigidity, headache and vomiting. Since non-polio EV infections are not mandatorily notifiable at federal level in Germany, no data on severe cases or deaths are available. No routine testing for EV is performed in deaths so we therefore cannot exclude fatal cases with other EV than EV-A71. Nevertheless, we were informed of an additional fatal case where EV-A71 was detected in a German child hospitalised in Austria in July 2019 (personal communication, Franz Allerberger and Birgit Prochazka, August 2019). Taken together with the fatal case reported in the EVSurv, this raises concern about increased occurrence of severe cases as a consequence of increased EV-A71 circulation.

Reports of severe clinical presentation associated with detection of a EV-A71 C1-like strains [3,5-7], together with recent indications of long-term sequelae [13,14], should be a signal for physicians and public health authorities to include/intensify EV diagnostic in patients showing signs of aseptic meningitis, encephalitis or acute flaccid paralysis/myelitis.

Continued virological data could provide first hints to upcoming public health matters. (EV typing should be conducted to enable molecular epidemiology. Furthermore, systematic studies are needed to assess the burden of EV infections in Europe.

## Laboratory Network for Enterovirus Diagnostic (LaNED)

Nikolaus Ackermann, Maja Adam, Armin Baillot, Ingrid Ehrhard, Martin Enders, Wali Hafezi, Ralph Hahn, Jörg Hofmann, Klaus Korn, Joachim Kühn, Detlef Michel, Alexandra Pettke, Christiane Prifert, Holger F. Rabenau, Ursula Reif, Gabriele Rieder, Benedikt Weißbrich, Andreas Wille and Marta J Zuchowski.
